# Chromatin Structure and Dynamics in Hot Environments: Architectural Proteins and DNA Topoisomerases of Thermophilic Archaea

**DOI:** 10.3390/ijms150917162

**Published:** 2014-09-25

**Authors:** Valeria Visone, Antonella Vettone, Mario Serpe, Anna Valenti, Giuseppe Perugino, Mosè Rossi, Maria Ciaramella

**Affiliations:** Institute of Biosciences and Bioresources, National Research Council of Italy, Naples 80131, Italy; E-Mails: valeria.visone@ibbr.cnr.it (V.V.); antonella.vettone@ibbr.cnr.it (A.Ve.); mario.serpe@ibbr.cnr.it (M.S.); anna.valenti@ibbr.cnr.it (A.Va.); giuseppe.perugino@ibbr.cnr.it (G.P.); mose.rossi@ibbr.cnr.it (M.R.)

**Keywords:** DNA topology, DNA structure, thermophilic organisms

## Abstract

In all organisms of the three living domains (Bacteria, Archaea, Eucarya) chromosome-associated proteins play a key role in genome functional organization. They not only compact and shape the genome structure, but also regulate its dynamics, which is essential to allow complex genome functions. Elucidation of chromatin composition and regulation is a critical issue in biology, because of the intimate connection of chromatin with all the essential information processes (transcription, replication, recombination, and repair). Chromatin proteins include architectural proteins and DNA topoisomerases, which regulate genome structure and remodelling at two hierarchical levels. This review is focussed on architectural proteins and topoisomerases from hyperthermophilic Archaea. In these organisms, which live at high environmental temperature (>80 °C <113 °C), chromatin proteins and modulation of the DNA secondary structure are concerned with the problem of DNA stabilization against heat denaturation while maintaining its metabolic activity.

## 1. Introduction

Chromatin associated proteins have the essential function of compacting, shaping and modelling the genome structure. Thanks to their combined action, the genome is organized into higher order, highly regulated and dynamic structures, which reduce its enormous length to fit into the nuclear or nucleoidal compartment, and make complex genome functions possible. Indeed, chromatin structure influences all information processes (transcription, replication, recombination and repair) and chromatin remodelling plays important regulatory roles in all these processes.

Chromatin proteins include architectural proteins and DNA topoisomerases. The first are small, basic DNA-interacting proteins generally not conserved at the primary sequence level, whose binding mode and structural effects on the genome are similar. They can induce DNA bending, looping, bridging or wrapping [1]. DNA topoisomerases are essential and evolutionary highly conserved enzymes inducing covalent modifications of DNA secondary structure and are responsible for the maintenance of proper DNA topology during the entire life of the cell [2,3,4]. Both classes of proteins contribute to maintenance and modulation of genome structure at two hierarchical levels, which affect each other; for example, the interaction between an architectural protein and its binding site on DNA can be regulated by DNA secondary structure and, conversely, DNA topoisomerases activity may be regulated by DNA binding proteins.

Chromatin structure studies, in particular in eukaryotes, have recently received great support thanks to the resolution of a number of 3D structures of these proteins and their complexes with DNA, as well as to the development of highly sophisticated methods employing atomic force microscopy, optical and magnetic tweezers, fluorescence imaging and chromatin sequencing. These techniques allow a wide range of analyses, from single-molecule up to genome-wide level, addressing the mechanisms and details of chromatin structure and function *in vitro* and *in vivo* (see for instance: [5,6,7,8,9]).

The Archaea comprise procaryotic microorganisms forming an evolutionary and functional domain distinct from Bacteria and Eukaryotes. Many archaeal species are characterized by peculiar and extreme habitats (hot springs, deep hydrothermal vents, saline and alkaline water, acid mines, antarctic ice, and so on). Although there is little knowledge of chromatin structure in Archaea, it is clear that their genomes are organized into a compact nucleoid. We here focus on architectural proteins and topoisomerases from hyperthermophilic archaea. In these organisms, which live at high environmental temperature (>80 °C <113 °C), chromatin proteins have the additional task of protecting DNA from denaturation while maintaining the flexibility needed to allow information processes. Most proteins and enzymes from these organisms show intrinsic high stability to heat and high thermophilicity, and generally their optimal temperature ranges are consistent with the growth temperature of their source. Some of them show the same activities as their mesophilic counterparts, but with higher thermal stability and higher ranges of temperature optima; other show very peculiar activities not found in proteins from other organisms. While we have tried to give a wide overview of the chromatin field in hyperthermophilic archaea and summarize main recent results, we are aware we could not cover many important aspects of this topic; we apologize to collegues whose work was not cited and direct readers to a number of excellent more specialized reviews [10,11,12,13,14,15,16].

## 2. Architectual Proteins of Hyperthermophilic Archaea

Archaea include at least two well-studied kingdoms, the Euryarchaea and Crenarchaea, as well as three other less well studied groupings, the Nanoarchaea, Korarchaea, and Thaumarchaea. Most studies on chromatin proteins have been performed on members of Euryarchaea and Crenarchaea, which show considerable diversity in chromatin-associated proteins: whereas Euryarchaea encode proteins similar to eukaryotic histones, most Crenarchaea typically do not, and instead contain a set of different architectural DNA-binding proteins [11,12,13]; some of these are shared by the two groups and have homologs in organisms outside the archaeal domain, but others are unique to one kingdom or even one genus (Table 1). This diversity is quite puzzling, also considering that information processing pathways show striking structural and functional conservation from archaea and eukaryotes.

**Table 1:** Distribution, main structural features and activities of architectural proteins of hyperthermophilic archaea. “√” and “-” indicate the presence or absence, respectively, of a particular activity or feature.

Protein	HISTONE	ALBA	SUL7	CREN7	SMJ12
Archaeal sub-domain	Euryarchaea and Crenarchaea	Euryarchaea and Crenarchaea	Crenarchaea (*Sulfolobus*)	Crenarchaea	Crenarchaea (*S. solfataricus*)
Oligomeric Structure	Dimer (7.5 kDa)	Dimer (10 kDa)	Monomer (7 kDa)	Monomer (7 kDa)	Dimer (12 kDa)
DNA Binding	Cooperative	Cooperative	-	-	-
DNA Modification	Compaction	√	-	√	√
	Bending	√	-	√	√
	Bridging	-	√	-	-
	Supercoiling	Negative	Negative	Negative	Negative
Post-translational modifications	NO	Acetylation/Deacetylation	Methylation	Methylation	unknown

### 2.1. Histones

**Figure 1:**
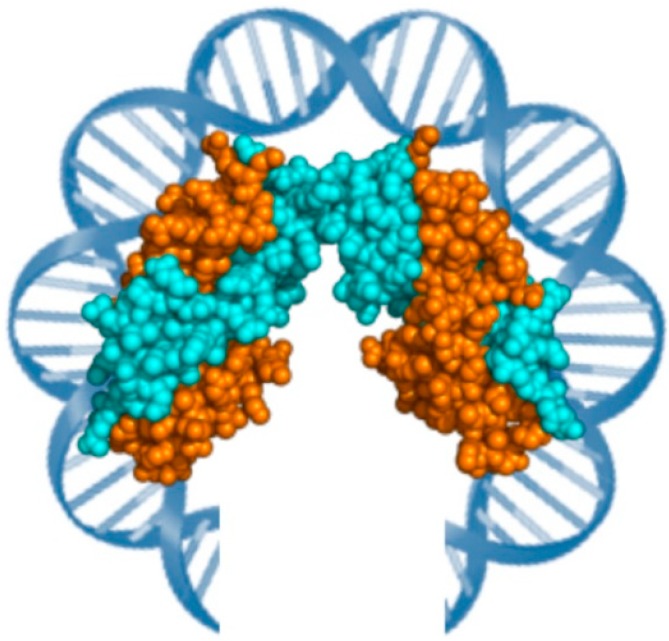
Structure of the *M. fervidus* histone tetramer in complex with DNA (PDB ID: 1B67), with the HmfA (orange) and HMfB (cyano) subunits.

Archaea belonging to the sub-domains *Euryarchaea*, *Nanoarchaea* and *Thaumarchaea* and some *Crenarchaea* encode homologs of eukaryotic histones [11,14,17,18,19,20,21]. Archaeal histones possess a typical fold resembling eukaryotic H3 and H4, consisting of three hydrophobic α-helices, and interact with the DNA minor groove. In solution, archaeal histones form dimers, whereas they bind DNA as tetramers or, less frequently, as hexamers [21,22,23]. *In vitro*, each histone tetramer protects about 60 bases of dsDNA; at low histone concentrations, DNA is sharply bent in complexes, suggesting wrapping of DNA around a histone tetramer [18,19,20,21]. Under these conditions, histone tetramers induce negative supercoiling of circular DNA molecules, whereas positive supercoiling is observed at non-physiological ionic strength [19,20,21]. Many Archaea encode multiple histone homologs; the most studied histone proteins from hyperthermophilic archaea are HMfA and HMfB from *Methanothermus fervidus.* These proteins can form either homodimers or heterodimers, which differ in their DNA binding properties and compact DNA into nucleosome-like structures [18,22] (Figure 1). Histone paralogs can be differently expressed in different growth phases and conditions, suggesting that the dynamic histone composition may shape chromosome structure differently [24,25].

Archaeal histones are considerably smaller than eukaryotic histones, due to the absence of the *C*- and *N*-terminal extensions that are targets of extensive post-translational regulatory modifications in eukaryotes [11]. No evidence for post-translational modification of archaeal histones has been observed, although protein acetyltransferase and methylase activities have been found in hyperthermophilic Archaea (see below).

Studies on eukaryotic chromatin established that nucleosomes are not positioned randomly in the genome, but rather different DNA segments facilitate nucleosome assembly depending on their primary sequence and the energy needed to wrap those fragments around the histones. In particular, alternating G/C- and A/T-rich dinucleotide tracts showed a propensity for histone-induced compaction; these observations led to the definition of a nucleosome positioning code [5]. Recent *in vitro* and *in vivo* experiments demonstrated that in the hyperthermophilic archaeal species *Methanothermobacter thermautotrophicus* and *Thermococcus kodakarensis* nucleosome assembly is directed by the same nucleosome positioning code observed in eukaryotes [26]. These results suggest that *in vivo* archaeal histones may use the same wrapping mechanisms as eukaryotic histones, although direct evidence is lacking. The chromatin organization of *T. kodakarensis* was also studied by applying a technology called chromatin particle spectrum analysis (CPSA), in which position and size of nucleosomal particles resistant to digestion by micrococcal nuclease were determined at the genomic level. This study demonstrated that *T. kodakarensis* chromatin particles consist of 30 bp units that can form linear multimers of variable length, up to ~450 bp. This structure is reminiscent of the so-called beads-on-a-string shape typical of eukaryotic chromatin; however, *T. kodakarensis* chromatin particles are in a dynamic equilibrium, in contrast to the static positioning of histones in eukaryotes. The 30-bp nucleosome units and their multimers were shown to colocalize with single or multiple, respectively, alternating G/C- A/T-rich dinucleotide tracts, a result consistent with the existence of a eucaryotic-type sequence preference code for nucleosome positioning in these organisms [27].

Another important issue is the relation between archaeal nucleosomes and gene expression. Several studies demonstrated that archaeal histones inhibit or reduce transcription by preventing preinitiation complex assembly and transcriptional initiation at promoters *in vitro*, suggesting that chromatin may play an important repressive function of basal archaeal gene expession *in vivo* [28,29]. Consistently, genome wide studies revealed that archaeal histones are excluded from genomic regions corresponding to transcription start sites, thus suggesting that, as in eukaryotes, promoters are nucleosome-free [26,27]. However, direct correlation between archaeal histone deposition and transcriptional status has not been established. Further studies are required to establish the exact structure of the nucleosomes in live archaeal cells and elucidate the relationships between nucleosomes and transcription. In particular, it would be interesting to assess whether the same chromatin structure and plasticity seen in *T. kodakarensis* are shared by all/other hyperthermophilic archaea; if this is the case, an attractive hypothesis is that chromatin plasticity might provide a mechanism to regulate gene expression by archaeal nucleosomes in the absence of the complex post-transcriptional control of eukaryotic histones.

### 2.2. Alba

Alba (Acetylation lowers binding affinity, reviewed in [30]) is a family of small, abundant DNA binding proteins (whose members are also known as Sac10 or Sso10, Ape10 *etc.*). These proteins are encoded by all thermophilic Archaea, most mesophilic Archaea and several eukaryotes [31,32,33]. In solution, Alba is a dimer of a 10-kDa subunit, which binds double-stranded DNA cooperatively without stringent sequence specificity and with high density (approximately 5 bp DNA per dimer), contacting the DNA minor groove; binding of Alba to DNA induces negative supercoiling, but not compaction [34,35,36,37].

Electron microscopy studies revealed that the binding of *S. solfataricus* Alba to DNA forms extended interwound helical protein fibres [34,38]. Alba binding has two effects on DNA, depending on its concentration: at low protein:DNA ratio the protein is able to bridge two DNA molecules, while at higher concentrations Alba dimers bind cooperatively along DNA molecules, increasing their rigidity; dimer–dimer interactions promote the cooperative binding, but also appear to be responsible for bridging DNA molecules together [32,39]. Alba-DNA interaction has been studied using single-molecule tethered particle motion and optical tweezers, confirming that Alba binds cooperatively, inducing a 5-fold increase in the persistence length of the nucleoprotein filament. Moreover, Alba concentration-dependent dimer–dimer contacts between two nucleoprotein filaments were also observed [40].

Some Archaea encode multiple Alba paralogs. For instance, *Sulfolobus solfataricus* encodes two Alba proteins; the more abundant Alba1 and the so-called Alba2, which is only 5%–10% of the Alba1 amount and has lower affinity for DNA. Alba2 forms obligate heterodimers with Alba1 at physiological concentrations [38]. Alba2 lacks the F60 residue that is responsible for the cooperative binding of Alba1 dimers; consistently, whereas Alba1 yields rigid protein–DNA complexes, at similar protein:DNA ratios Alba1:Alba2 heterodimers form condensed protein–DNA complexes [39]. These results were confirmed by single-molecule techniques [41]. The dual binding mode of Alba and the existence of multiple Alba paralogs with different DNA binding properties suggest that these proteins are well-suited to play an important role in modelling chromatin structure by regulating the equilibrium between stiff and interlinked DNA. Chromatin immunoprecipitation experiments showed that *S. solfataricus* Alba1 is widely distributed at many loci along the genome, thus supporting its role in chromosomal organization [42]. In particular, the ability of Alba to bridge DNA molecules suggests that it might participate in organizing the genome into higher order loops, as those found in bacteria, although the existence of such structures in archaeal genomes has not been proven to date.

**Figure 2:**
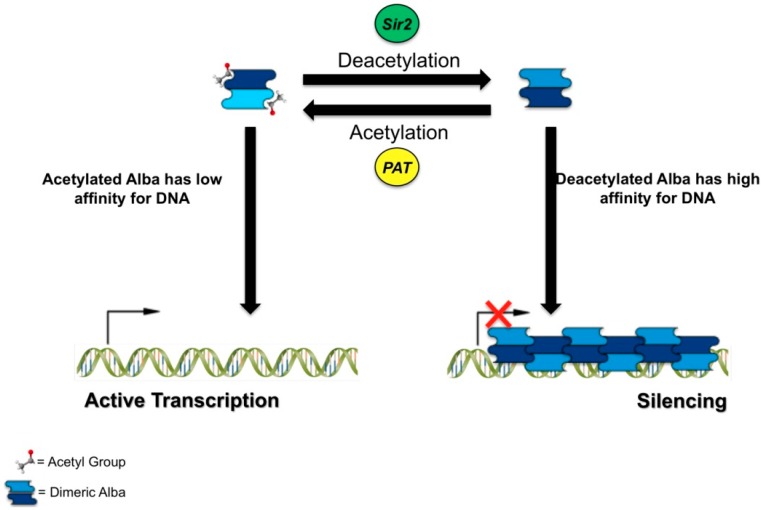
Diagram showing a model for the post-translational regulation of Alba and its effect on trancription.

A breakthrough in the archaeal chromatin field was the discovery that in *S. solfataricus* cells Alba1 interacts with the archaeal homolog of the eukaryotic silencing protein Sir2 and this interaction regulates Alba1 DNA binding affinity [31]. *In vivo*, a significant fraction of *S. solfataricus* Alba1 protein is found specifically acetylated at the lysine 16 residue and this modification is reversed *in vitro* by the deacetylase activity of Sir2. Acetylation determines a significant decrease of the affinity of Alba1 for DNA. Using *in vitro* systems Alba1 was shown to have a repressive effect on transcription; interestingly, the efficiency of transcriptional silencing correlates with Alba1 modification: the acetylated form is about three-fold less effective than the Sir2-deacetylated form, which is consistent with the relative affinity for DNA of the two protein forms (Figure 2) [31].

Alba1 lysine 16 acetylation is specifically catalysed by an acetyltransferase called PAT, which is conserved not only in archaea, but also in bacteria, although in this latter it seems to play a metabolic role [42,43]. Incubation of Alba1 with PAT reduces Alba1 affinity for DNA and this effect requires the presence of an acetyl-donor, thus confirming that PAT-mediated acetylation reduces Alba1 affinity for DNA. Although no data are available on the effect of Alba1 acetylation and Alba1-Sir2 interaction *in vivo*, an attractive model is that these proteins play a role not only in chromosome structure, but also in transcription regulation. Sir2-induced Alba deacetylation would stimulate recruitment of Alba to DNA (and possibly further deacetylation of other Alba molecules) resulting in spreading of a transcription repressive state due to Alba binding (Figure 2). Although in principle a similar model might be applied to other organisms encoding homologs of both Alba and Sir2, from archaea to higher eukaryotes, the general relevance of these findings is not yet clear. The functional interaction between Alba and Sir2 and the effect of Alba on transcription is conserved the malaria protozoan *Plasmodium falciparum* [44]. However, the Lys-16 residue is not conserved within the Alba protein family, raising the possibility that in some members surrogate acetylated residues exist, which need to be identified to fully understand Alba’s role in chromatin regulation.

The crystal and solution structures of Alba proteins from several archaeal species have been resolved [32,38,45,46,47,48,49,50]. These structures revealed that Alba shows a fold similar to that of the *N*-terminal domain of DNAseI and the *C*-terminal domain of bacterial translation factor IF3. The structural similarity to this latter factor, an RNA-binding protein, suggested that Alba may also have some RNA-related function, a hypothesis supported by the observation that Alba can bind to RNA *in vivo* and *in vitro* [51]. However, if this hypothesis is correct, such a function remains to be addressed. The 3D structure revealed that the protein forms a dimer of dimers, in which the dimer–dimer interface is stabilized by several hydrophobic residues centered around a phenylalanine (F60 in *S. solfataricus* Alba1) critical for dimerization [39,46]. Interestingly, the lysine residue corresponding to the *S. solfataricus* Alba1 K16, which is the target of the regulatory PAT-induced acetylation and Sir2-dependent deacetylation, is also involved in inter-dimer interaction, thus suggesting an elegant model linking the effect of Sir2-induced deacetylation to Alba oligomerization and DNA binding efficiency [38,39,46].

Resolution of an Alba2-DNA complex showed that each protein dimer contacts the minor groove and covers 4 DNA bases [49]. Binding induces a conformational rearrangement of the protein which facilitates protein oligomerization. The two types of dimer–dimer interactions were also observed in the DNA-bound structures; whereas interactions between adjacent dimers are mediated by chains of hydrogen bonds, the dimer–dimer interface between two DNA–protein fibers is stabilized mainly by interaction between conserved hydrophobic residues [49]. In all complexes, DNA was in its extended form, leaving the question of the role of Alba in DNA condensation still open. It is possible that *in vivo* the cooperation with other factors (such as histones, Sul7, Cren7, or analogous proteins) is needed to achieve appropriate compaction level. Thus, it would be interesting to analyse the physical and functional interaction of Alba with other chromatin proteins.

In eukaryotes chromatin activities, including its repressive effects on transcription, are modulated by post-translational histone modifications, such as acetylation, methylation and phosphorylation. Interestingly, the crystal structure of the Alba acetylase PAT in complex with coenzyme A revealed structure similarity to eukaryotic histone acetyltransferases, suggesting an intriguing analogy between Alba and eukaryotic histones [43]. However, in contrast to Alba, whose deacetylation induces trascriptional silencing, eukaryotic histone acetylation determines transcription activation, although the mechanism of acetylation-dependent transcription regulation is not completely clear. An intriguing hypothesis is that eukaryotic histones acetylation regulates transcriptional activity with a mechanism similar to Alba, although with opposite effects, *i.e.*, acetylation of histone tails may disrupt intermolecular interactions in higher order chromatin structures [46]. Confirmation of this model would require further studies on archaeal as well as eukaryotic chromatin proteins.

### 2.3. Sul7 and Cren7

Sul7 (formerly known as Sso7d, Sac7d or Ssh7d, reviewed in [1,13]) is a 7-kDa basic, abundant, non-specific DNA-binding protein found only in Crenarchaea of the genus *Sulfolobus*, where it accounts for 3%–5% of total protein content and binds strongly to double-stranded DNA without sequence preference. Binding protects DNA from thermal denaturation, elevating the melting point by 30 °C [52]. Moreover, it promotes the renaturation of complementary DNA strands at temperatures higher than the melting point of the duplex [53]. The annealing activity, which is strictly homology-dependent, might assist renaturation of the double helix at high temperature in processes requiring transient denaturation, such as transcription, recombination and repair. On the basis of these findings it can be suggested that Sul7 plays a key role in stabilization of DNA at high temperature.

The 3-D structure of Sul7, solved both by NMR and X-ray crystallography [52,54,55,56] showed that the protein consists of two orthogonal anti-parallel β-sheets (one triple- and one double-stranded). This folding is reminiscent of that of the SH3 domain found in several eukaryotic proteins. Crystal structure of Sul7-DNA complex showed that it binds the DNA minor groove and induces changes in the helical twist and marked DNA bending (60°). These observations have been confirmed by *in vitro* functional assays, showing that Sul7 induces bending of short DNA fragments and compaction of circular DNA molecules of any topology (negative, relaxed, or positive [57]).

Sul7 induces negative supercoiling of DNA in association with DNA topoisomerases. In particular Sul7 and the *Sulfolobus* DNA topoisomerase TopoVI (see below) induce negative supercoiling of circular DNA molecules at physiological temperatures (up to 80 °C), transforming the conformational changes induced by Sul7 into topological changes [57]. These results suggest that Sul7 plays a role in DNA packaging and in the regulation of DNA superhelicity in *S. solfataricus*. Moreover, Sul7 inhibits the positive supercoiling activity of reverse gyrase (see below), probably by stabilizing the double strand and inhibiting transient exposure of single strand regions required for reverse gyrase binding (see below). Indeed, Sul7-induced reverse gyrase inhibition is antagonized by the single strand binding protein, SSB, suggesting a functional interplay among Sul7, reverse gyrase and SSB in a physiological context [58].

Experiments *in vivo* showed that in *S. solfataricus* *Sul7* gene expression as well as the protein localization are affected by cell exposure to DNA damage: the *Sul7* gene transcription is repressed after cell exposure to UV light [59], whereas the protein dissociates from chromatin after treatment with alkylating agents [60].

Cren7 is also a small (about 7 kDa) monomeric, abundant chromatin protein conserved amongst hyperthermophilic crenarchaea [61]. It is similar in many respects to Sul7, although they are different at primary sequence level. Indeed, Cren7 binds the minor groove of DNA non-specifically and the DNA interacting surface is a triple-stranded β-sheet. 3D structures of Cren7 and its complex with dsDNA showed that it shares the same SH3-like fold already found in Sul7. Upon binding, Cren7 binds compacts and kinks the dsDNA sharply, constrains negative DNA supercoils *in vitro* and is associated with genomic DNA *in vivo*. Molecular dynamics simulations at different temperatures indicate that Cren7 stabilizes the DNA duplex, while DNA molecules undergo B-like to A-like form transitions with increasing temperature [62].

For their functional and structural similarities, Sul7 and Cren7 could be both involved in genome packaging; although direct *in vivo* evidence is lacking, the fact that they are encoded by organisms (Crenarchaea) lacking histones makes this assumption likely. However, it should be noted that, whereas Cren 7 is present in all Crenarchaea, Sul7 is only found in the genus *Sulfolobus*, raising the question of the functional relationship and redundancy of the two proteins in different archaeal strains. In this respect, some significant differences in the structure of the two protein-DNA complexes and their *in vitro* activity have been found. Cren7 contains a large loop in the DNA binding surface, which is lacking in Sul7; this loop contains residues important for DNA binding of Cren7 [63,64], including one lysine residue, which undergoes reversible methylation [61] (see also below). Moreover, Cren7, but not Sul7, shows a *N*-terminal tail comprising serine and lysine residues; although evidence is lacking, an intriguing hypothesis is that these residues are targets of post-translational modifications, like in eukaryotic histones. The binding site of Cren7 is larger than that of Sul7 (8 *vs.* 4 bp), and biochemical experiments showed that Cren7 is twice as efficient as Sul7d in constraining negative supercoils [61], although this conclusion has been recently challenged by results obtained by combining atomic force microscopy and magnetic tweezers with molecular dynamics studies. These experiments demonstrated that the interaction of the two proteins with DNA is similar, as their binding affinity and extent of DNA compaction [65]. Moreover, Cren7- and Sul7-DNA complexes differ in flexibility from analogous bacterial and eukaryotic DNA-bending proteins [65].

Interestingly, both Sul7 and Cren7 are found to be monomethylated *in vivo* at specific lysine residues; for Cren7, these residues are located in the flexible loop close to the DNA-interacting surface [61], whereas the five Sul7 lysine residues found specifically methylated *in vivo* are not involved in DNA binding [52]. Consistently, lysine methylation affects the DNA binding affinity of Cren7 but not Sul7d [61]. Recent work identified a lysine methyltransferase called aKMT4 as a candidate factor responsible for this post-translational modification [66,67]. aKMt4 is well conserved in the genomes of Crenarchaea and shows striking structural and functional similarity to the eukaryotic histone methyltransferase KMT4/Dot1. Sul7 and Cren7 are both substrates of aKMT4-induced methylation *in vitro.* Methylation of Sul7, but not Cren7, is significantly stimulated by the presence of DNA; in particular, the level and efficiency of Sul7 methylation by aKMT4 are increased by pre-incubation of the protein with DNA. Since Sul7 methylation *in vivo* occurs only at lysine residues not involved in DNA binding, the results of the *in vitro* experiments suggest that aKMT4-induced methylation might occur on the chromatin-bound Sul7, and a possible regulation of aKMT4 activity by the local chromatin environment [67]. Whereas methylation in the flexible loop might regulate the Cren7 DNA binding affinity in chromatin the functional significance of this modification remains to be elucidated for Sul7. The extent of Sul7 lysine methylation increases *in vivo* with increasing growth temperature, suggesting a heat-shock response related functional relevance [52]. These observations suggest that the Sul7 and Cren7-DNA interaction might be regulated differently, leading to the speculation that they may also have distinct functions; for instance, they may regulate dynamically chromosomal organization in response to different metabolic or environment conditions, or control the access of different proteins to chromatin. However, no data are available to support such hypotheses and further studies are required to address these points.

It is interesting to note that, in contrast to archaeal histones, three archaeal architectural proteins, Alba, Sul7 and Cren7 are all subject to post-translational modifications (acetylation or methylation). Extensive post-translational modifications play essential roles in establishing the epigenetic regulation of eucaryotic histones. Thus, post-translational modification of chromatin proteins represents an ancient and evolutionary conserved model for regulation of chromatin accessibility.

Sul7 was demonstrated to inhibit the activity of the DNA topoisomerase reverse gyrase [57] (see below); moreover, both Sul7 and Cren7 were shown to inhibit the DNA polymerase B1 from *S. solfataricus* in its strand displacement activity, which is likely involved in Okazaki fragment maturation during replication. Sul7 and Cren7 inhibit the polymerase ability to displace DNA–DNA, but not DNA–RNA hybrids, thus suggesting that the chromosomal proteins might take part in this process, directing the polymerase activity to removal of RNA primers while inhibiting extensive displacement of the newly synthesized DNA strand [68].

### 2.4. Other Architectural Proteins

Another protein affecting DNA conformation is Smj12 [69]. Smj12 is a member of the so-called BA (Bacterial-Archaeal) family, a large family of Helix-Turn-Helix DNA-binding proteins widespread in Archaea, and shares significant aminoacid identity with the transcriptional repressor Lrs14 [70]. Smj12 is a 12 kDa very basic protein, dimeric in solution and highly thermostable. Smj12 is a non-specific DNA binding protein that protects double-stranded DNA from thermal denaturation. Unlikely Sul7 or Cren7, Smj12 does not compact DNA or induce bending *in vitro*, rather it induces positive supercoiling of DNA in topological assays. *In vivo* it is far less abundant than either Sul7 or Alba, suggesting that it is unlikely to organize higher order structures over the whole chromosome, but rather might have more specific functions [69].

CC1 is a small basic protein found only in a few Crenarchaea, whose function has not been elucidated. CC1 is a 6-kDa, monomeric, basic protein that is expressed at a high level in *Thermoproteus tenax*. It resembles Sul7 and Cren7 for its β-sheet rich structural organization, although it does not share amino acid sequence similarity with these proteins. Moreover, in contrast to both Sul7 and Cren7, CC1 binds both ssDNA and dsDNA with comparable affinity, although binding to ssDNA is highly cooperative [71].

## 3. DNA Topoisomerases from Hyperthermophilic Archaea

DNA topology is controlled and maintained by the action of DNA topoisomerases, essential enzymes that regulate the supercoiling level of DNA during all DNA activities (replication, transcription, recombination and repair). DNA supercoiling serves not only an important role in DNA compaction, but also regulates protein–DNA interactions and affects DNA transactions. DNA topoisomerases are classified according to their structure, specific activity and cellular function [2,3,4,10] (Table 2). DNA topoisomerases of Type I are monomeric, ATP-independent enzymes which induce a transient break in one DNA strand and pass the other intact strand through this “hole”, leading to DNA relaxation. The type I topoisomerase family comprises three sub-types, which differ in both structure and activity: Type IA topoisomerases relax only negative supercoils and are found in Bacteria, Eukaryotes and Archaea, whereas Type IB enzymes relax both negative and positive supercoils and are mostly eukaryotic. The type C sub-family comprises at the moment only one member from a hyperthermophilic archaeon (see below). Type II topoisomerases are heteromeric enzymes (dimer or tetramers), induce transient breaks of both DNA strands and relax both positive and negative supercoiling at the expense of ATP hydrolysis. Also for the TopoII family two sub-types (A and B) have been found so far, which differ significantly in their structure and action mechanism. Type IIA topoisomerases are ubiquitous in Bacteria and Eukarya, whereas members of the IIB family are mostly present in Archaea and plants.

**Table 2:** Features of DNA topoisomerases of hyperthermophilic archaea. Type IA enzymes are shared by almost all archaea, bacteria and eukaryotes; type IIB are present in all archaea and some plants; reverse gyrase is found only in hypethermophilic archea and bacteria; type IC is restricted to *M. kandleri*.

Enzyme	Topo VI	Topo 3	Reverse Gyrase	Topo V
Type	II B	IA	IA	IC
Structure	Heterotetramer A_2_ + B_2_ (A domain containing Winged Helix Domain (Active Site)/B domain containing ATP Binding Site)	Monomer (*C*-terminal domain implicate in DNA binding/*N*-terminal domain implicate in topoisomerase activity)	Monomer (*C*-terminal domain like Topoisomerases type IA/*N*-terminal domain like SF2 helicases)	Monomer (*N*-terminal domain with topoisomerase activity/*C*-terminal domain with AP site processing activity)
Activity	ATP dependent positive and negative supercoiled DNA relaxation; Cut double strand	ATP independent negative supercoiled DNA relaxation; Cut single strand	ATP dependent positive supercoiled DNA; ATP independent negative supercoiled DNA relaxation; Cut single strand	Positive and negative supercoiled DNA relaxation; Cut single strand

**Figure 3:**
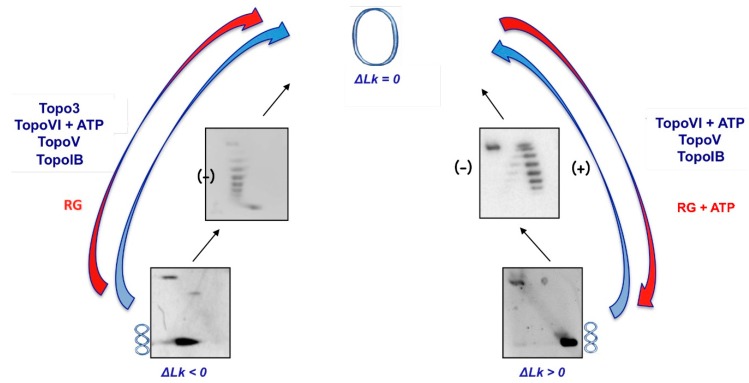
Activities of DNA topoisomerases of hyperthermophilic archaea on circular plasmids. The gels show typical migration of plasmid topological isomers: Δ*Lk* (Linking number) < 0 (negatively supercoiled); Δ*Lk* = 0 (relaxed); Δ*Lk* > 0 (positively supercoiled). Red arrows indicate the direction of activity of reverse gyrase (RG), which is always directed toward increasing linking number (from negative, to relaxed, to positive topoisomers). Blue arrows indicate the activity of all other topoisomerases, e.g., relaxation of either negative or positive topoisomers, with the exception of Topo3, which only relaxes negative supercoiling.

The topological state of chromosomal DNA in hyperthermophilic archaea is not clear, although several lines of evidence suggest that it is relaxed or positively supercoiled [15,16] and thus likely more resistant to thermal denaturation as compared with the negatively supercoiled DNA of bacteria. This peculiar conformation results from the concerted activity and balance of a number of DNA topoisomerases of different families, including one type IA (Topoisomerase 3), one type IIB (Topoisomerase VI), and two peculiar chimeric enzymes, the type IC/AP lyase Topoisomerase V and the type IA/helicase reverse gyrase (Figure 3) [10].

### 3.1. Topoisomerase VI

DNA Topoisomerase VI (TopoVI) [72,73] is classified as a Type IIB enzyme. Indeed, TopoVI is capable of cleaving both DNA strands, catalysing the passage of a DNA duplex through the double-strand break and religating the product resulting in relaxation of either negative or positive topological stress. The reaction is strictly ATP-dependent. However, whereas TopoIIA cleavage leaves a four base overhang, there are only two after Topo VI cleavage [74]. TopoVI is a heterotetramer comprising two A and two B subunits, each encoded by a separate gene. Despite the similar organization, with the tyrosine responsible for DNA cleavage in the A subunit and the ATP binding site in the B subunit, TopoVI shares limited similarities with type IIA topoisomerases. As in TopoIIA enzymes, the TopoVI B subunit contains an ATP-binding site located within a protein domain known as the Bergerat fold, which is also found in other ATPases [73,75]. Structural analysis has shown that upon ATP binding and hydrolysis the monomeric B subunit undergoes dramatic conformational changes leading to dimerization [76]. In contrast, the TopoVI A subunit is completely different from the one of TopoIIA enzymes [77], whereas it is homologous to the eukaryotic meiosis-specific phosphodiesterase Spo11 [73]. Interestingly, TopoVI shares with TopoIIA enzymes the so-called Toprim domain, involved in magnesium binding; however, whereas in type IIA topoisomerases the Toprim domain is located in the B subunit, it is located in the A subunit of TopoVI. Lastly, both enzyme classes show a Winged Helix (WH) domain containing the active site tyrosine, although these regions are different in their primary sequence.

The two available complete structures of TopoVI showed the so-called “twin-gate” architecture, also found in TopoIIA enzymes, in which the ATPase domains of the two B subunits, located at one side of the heterotetramer, are able to coordinate DNA sliding through the DNA cleaving sites located at the opposite side of the molecule. This is possible due to conformational changes occurring during the reaction that couple nucleotide hydrolysis with strand passage [78,79]. Thus, despite the lack of conservation at the primary sequence level, TopoII A and B share significant structural similarity and reaction mechanism.

### 3.2. Topoisomerase 3

Archaeal Topoisomerase 3 (Topo3) is a canonical Type IA DNA topoisomerase. These enzymes are present in all organisms, with a few exceptions [80] and catalyze the relaxation of negatively, but not positively supercoiled substrates; this reaction does not require nucleotide hydrolysis. Experiments performed in many different organisms showed clearly that Topo3 enzymes play many roles in the cells, including regulation of the supercoiling level, and are involved in transcription, recombination and repair (see below). They are widespread in hyperthermophilic archaea, although only a few members have been studied. Topo3 from *S. solfataricus* (SsTop3), consistent with its hyperthermophilic source, relaxes negative supercoiling and works optimally at 75 °C; however, ssDNA cleavage occurs even at lower temperatures (25–50 °C), whereas ligation of the cleaved DNA requires temperatures higher than 45 °C. In addition, SsTop3 induces efficient annealing of complementary ssDNA, an activity not shared by all Topo3 enzymes; annealing may participate in the catalytic cycle, stimulating religation of the DNA strand [81,82].

Deletion of Topo3 gene in *Sulfolobus islandicus* is not lethal, although the mutant growth rate is retarded with respect to the wild-type strain, in particular under nutrient deprivation conditions. The knock-out mutant shows alterations in the cell cycle, a high frequency of impaired DNA segregation and cell division and significant changes in the global transcriptional profile [83]. Taken together, these results suggest that archaeal Topo3 may be involved in chromosomal segregation and regulation gene expression through control of the level of intracellular DNA supercoiling.

More recently, a peculiar Topo3 enzyme from the endoparasitic hyperthermophile *Nanoarchaeum equitans* (NeqTop3) has been identified. In contrast to other topoisomerases of this family, NeqTopo3 is an heterodimer consisting of two polypeptides encoded by a split gene. Besides the classical relaxation of negatively supercoiled DNA, this enzyme was shown to also catalyze a distinct reaction, the synthesis and dissolution of hemicatenanes. This reaction is due to DNA strand passage through denatured bubbles in the substrate DNA, which can be transiently exposed at the high temperature of incubation. At lower NeqTop3 concentrations, hemicatenanes are removed [84].

In both bacteria and eukaryotes, Topo3 enzymes interact physically and/or functionally with RecQ family helicases, and these complexes play multiple roles in recombination, repair, replication, and transcription. Analogously, SsTop3 was shown to interact with an archaeal RecQ-like helicase, Hel112. This enzyme shares limited sequence homology with eukaryotic RecQ helicases, but catalyses similar activities, including coordinate DNA unwinding and annealing, processing of synthetic Holliday junctions and stabilization of model replication forks [85,86]. SsTop3 inhibits the Hel112 helicase activity and stimulates formation and stabilization of Holliday junctions. The interplay between Hel112 and SsTop3 might regulate the equilibrium between recombination and anti-recombination activities at replication forks [86].

### 3.3. Reverse Gyrase

Reverse gyrase is an enzyme with peculiar structure and function. It consists of a Type IA topoisomerase module fused to a SF2 helicase–like domain, and induces ATP-dependent positive supercoiling of DNA; the reaction requires high temperature (>60 °C) [87,88,89,90,91,92]. Reverse gyrase is considered a thermophile-specific marker, since its gene is invariably present in the genomes of all bacteria and archaea living above 80 °C (and in some living at intermediate temperatures) but in no mesophilic organisms [93,94,95]. Positive DNA supercoiling increases the resistance of DNA to denaturation and consistently, plasmids isolated from hyperthermophiles show higher linking number as compared with plasmids from mesophiles [15,16]. Moreover, reverse gyrase protects DNA incubated at high temperature from depurination and degradation [96]. Based on these observations, a role for reverse gyrase in adaptation to high temperature has long been suggested; however, direct evidence of such a role is still lacking. Genetic experiments showed that deletion of the single reverse gyrase gene in the species *T. kodakarensis* is not lethal [97], although the mutant is less thermoresistant than the wild-type. In contrast, although *S. islandicus* encodes two reverse gyrase genes, neither could be deleted, suggesting that both are essential [98]. Certainly, convincing experiments in other species are required to ascertain the role of reverse gyrase in adaptation to high temperature.

The 3D structure of two reverse gyrases has been resolved, only one of which is from an archaeon, *Archaeoglobus fulgidus* [99]. Comparison of this structure with that of the enzyme from the bacterium *Thermotoga maritima* [100] revealed a well conserved type IA topoisomerase fold for the *C*-terminal domain, and a less conserved fold for the *N*-terminal domain, which contains a typical ATP-binding fold resembling that of the SF2 family helicases.

Mutational analysis of several archaeal reverse gyrases has identified motifs essential for DNA topoisomerase, ATPase and DNA binding activity [101,102,103,104] as well as the role of the so-called latch sub-domain, a region connecting the ATPase and topoisomerase modules [105,106].

Significant diversity in the details and optimal conditions of the reaction is seen among different archaeal reverse gyrases: their temperature optima range from 75 to 95 °C and ionic strength tolerance from 400 and up to 1200 mM; all RGs require a nucleotide (preferably ATP) for positive supercoiling reaction, however, in the absence of the nucleotide some, but not all, RGs are able to relax DNA; some enzymes are very processive, whereas other show a distributive behaviour of their positive supercoiling reaction [99,101,102,103,104,105,106,107]. The recently characterized reverse gyrase from *Pyrobaculum calidifontis* (PcalRG) [104] shows even remarkable thermostability, with significant activity even at 100 °C, and DNA binding and topoisomerase activity up to 1.2 M NaCl. The structural bases of these differences are not clear.

Given its chimeric helicase-topoisomerase structure, a long-standing question is whether there is any functional analogy between reverse gyrase and RecQ–Topo3 complexes (reviewed in [108]). The *in vitro* activity of such complexes from a number of mesophilic bacteria and eukaryotes have been characterized, showing that they can use coordinate DNA unwinding, annealing and topoisomerase activities to catalyse complex reactions, such as reversal of replication fork, branch migration and resolution of model recombination intermediates [109,110]. The structural similarity of reverse gyrase with RecQ–Topo3 complexes stimulated a wave of studies to establish whether they are also functionally similar. One set of experiments was aimed at testing whether RecQ–Topo3 complexes may show positive supercoiling activity: co-incubation of human RecQ1 and Topo3α or *E. coli* RecQ and Topo3 failed to catalyse positive supercoiling at mesophilic temperature [111]; these observations, however, are of uncertain significance, since even reverse gyrase (the only topoisomerase shown to induce positive supercoiling) is not able to do so below 60 °C. Recently, the availability of thermophilic SsTop3 and Hel112 allowed the direct comparison of the activities of this complex with those of reverse gyrase. Although the SsTop3–Hel112 complex shares some activities with reverse gyrase on oligonucleotide substrates (see below), it was unable to induce positive supercoiling at high temperature under the same conditions that allow reverse gyrase to catalyse this reaction [86]. Thus, positive supercoiling is a peculiar activity of reverse gyrase and not a basic property of helicase-topoisomerase complexes.

The second set of experiments was aimed at testing whether reverse gyrase shows DNA unwinding, annealing and branch migration activities typical of RecQ and RecQ–Topo3 complexes. In earlier studies using classical helicase assays reverse gyrase failed to show processive DNA unwinding activity [101,102,112]. However, one of the two *S. solfataricus* reverse gyrases was shown to destabilize *in vitro* synthetic substrates resembling Holliday junctions (HJ) [113]. In this reaction, the enzyme does not act as a processive helicase; indeed, the reaction does not require the presence of ATP or a functional ATPase activity; moreover, mutational analysis showed that the topoisomerase activity is also dispensable. The enzyme binds at the core of the HJ, inducing a structural distortion that likely facilitates junction unfolding at high temperature.

**Figure 4:**
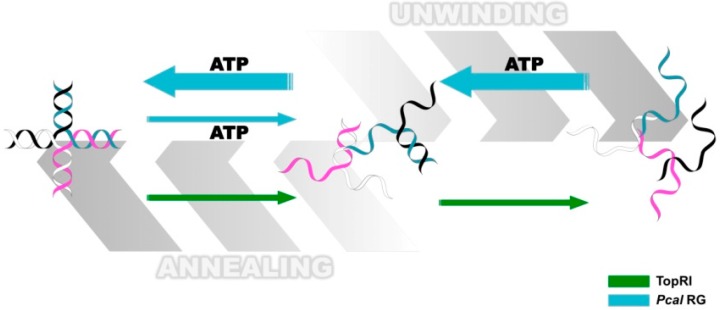
Diagram comparing the activities of the *S. solfataricus* TopR1 and *P. calidifontis* PcalRG reverse gyrases on synthetic Holliday junctions. Light blue arrows indicate PcalRG activities (ATP-dependent unwinding and ATP-indepedent annealing); green arrows indicate TopR1 ATP-independent unwinding; the four DNA strands are shown in white, pink, black and blue, respectively. Whereas TopR1 unwinding activity proceeds up to single strand oligonucleotides, the main products of PcalRG unwinding are Y-shaped forks, likely because single strand products are annealed back [104,113].

More recently, it was reported that PcalRG shows a real helicase activity, namely ATP-hydrolysis dependent unwinding of ds DNA and HJ structures [104]; interestingly, at higher enzyme/DNA ratios the reaction is reversed leading to re-annealing of DNA (Figure 4).

Although PcalRG is the only reverse gyrase for which processive ATP-dependent unwinding and annealing activities have been demonstrated, it is possible that these abilities are shared by other reverse gyrases. Indeed, the reverse gyrase from the bacterium *T. maritima* was shown to induce transient unwinding of a short DNA duplex; the short-lived unwound intermediate does not accumulate because the reaction is rapidly reversed in the presence of ATP, and can only be seen using an ATP analog [114]. Combined unwinding and annealing activities could help clarify the mechanism of the positive supercoiling reaction. Indeed, unwinding of a DNA region in a topologically closed molecule would create two domains, one positive and one negative; a topoisomerase IA activity capable of relaxing only this latter would result in positive supercoiling of the final product [115,116].

The importance of DNA supercoiling modulation by reverse gyrase is confirmed by the involvement of the enzyme in the cell response to DNA damage and its interaction with chromatin and repair proteins [57,58,60,117,118]. In *S. solfataricus* reverse gyrase is recruited to chromatin after UV irradiation [96]; moreover, it forms a complex with the single-strand binding protein, SSB and the translesion DNA polymerase, PolY [118]. Interestingly, reverse gyrase inhibits PolY activity, and inhibition depends on the positive supercoiling activity of reverse gyrase. These results suggested the hypothesis that PolY might be sequestered onto highly positively supercoiled regions of the genome when its activity is not required, and illustrate an example of how the interaction with a chromatin protein might provide a means to control the potentially mutagenic activity of PolY under normal growth conditions.

Reverse gyrase also interacts with Sul7, and Sul7 inhibits reverse gyrase positive supercoiling activity [57]; since Sul7 induces negative supercoiling (see above) it is possible that the interplay between these proteins with antagonizing effect in the context of chromatin might contribute to control of the homeostasis of supercoiling level.

### 3.4. Topoisomerase V

Another peculiar archaeal topoisomerase is Topoisomerase V (TopoV), an enzyme so far found only in the species *Methanopyrus kandleri*. Although it belongs to the type I family of topoisomerases, TopoV is unrelated to either type IA or IB enzymes. Like eukaryotic Topo IB enzymes, it is able to relax both positively and negatively supercoiled DNA forming a 3'-link with DNA [119]; the enzyme is extremely thermophilic (being active up to 122 °C) [120]. TopoV is unique among all known topoisomerases in its structure combining a topoisomerase and a DNA repair domain. The DNA topoisomerase module is located at *N*-terminus of the protein, whereas the *C*-terminal domain exhibits an apurinic/apyrimidinic site-processing activity (AP lyase) [121]. This latter domain contains 24 helix-hairpin-helix repeats which were shown to contribute to the high salt resistance and processivity of TopoV [121,122]. Based on these peculiarities, Forterre [123] assigned TopoV to a third family of type I DNA topoisomerases, named Topo IC; he also hypothesised that this enzyme, like many other orphan proteins, could have a viral origin.

The crystal structure of the 61 kDa *N*-terminal fragment of TopoV revealed no structural similarity to other topoisomerases. In particular, the structure of the active site region is unique, suggesting no conservation in the cleavage and religation mechanism. Moreover, the active site is not exposed on the molecule surface, suggesting the need for extensive conformational changes during the catalytic cycle [124]. By using real-time single-molecule and micromechanical experiments TopoV was shown to relax DNA using a constrained swiveling mechanism, similarly to type IB enzymes, and to perform multiple DNA relaxation cycles before discharging from DNA. Relaxation efficiency is enhanced by DNA supercoiling, but is reduced by the contacts between protein and DNA. Thus, TopoV relaxes DNA using a similar overall mechanism as type IB molecules, but in a completely different structural context [125]; an interesting line of future studies would be whether TopoV might also accomplish the same functions as the eukaryotic TopoIB. Recently, structural and biochemical studies demonstrated that an *N*-terminal 69-kDa fragment of TopoV is the minimal fragment with both topoisomerase and AP lyase activities and a putative Lys residue involved in the AP lyase activity was identified [126].

### 3.5. Topoisomerase IB

Topoisomerase IB (TopoIB) was thought for a long time to be a eukaryotic specific enzyme. However, recently a TopoIB-like gene was found in the sequenced genomes of two archaea of the phylum Thaumarchaeota. Phylogenetic analyses suggest that a TopoIB-like gene was present in the last common ancestor of Archaea and Eucarya [127]. The function of this archaeal TopoIB is currently unknown.

## 4. Conclusions

Whereas a large amount of data on architectural proteins and DNA topoisomerases of hyperthermophilic archaea has been accumulated over the last years, much work is still needed to delineate a complete picture of chromatin structure and regulation in these organisms. Since most information on this topic has been gained using *in vitro* assays, one of the current main limitations in the field is the lack of *in vivo* data. This has been due in part to difficulties in the cultivation of some hyperthermophilic archaea species under laboratory conditions, and to technical setbacks that only recently allowed genetic manipulation of a few species, and still hamper it for the majority of these organisms. Hopefully, advances in genetic and cell biology techniques for hyperthermophilic archaea will help fill the gap in the near future. In addition, many questions that need to be addressed concern the relationships among different chromatin proteins, and in particular of members of the Alba family, which is present in all hyperthermophilic archaea, and other architectural proteins, namely histones in euryarchaea and Cren7 (and Sul7) in crenarchaea. In general, data on chromatin protein–protein interactions are scarce; a complete map of the interactions of chromatin proteins with each other and with other cellular proteins will help elucidate the role of chromatin in information processes and eventually lead to the identification of chromatin remodelling factors in hyperthermophilic archaea, a still uncovered area.

Another important issue is the role of reverse gyrase in cell resistance to high temperature; the hypothesis that the enzyme is an important factor in this adaptation is tantalizing given the restriction of reverse gyrase to hyperthermophiles, but experiments on different organisms are required. Moreover, still new functions for DNA topology and topoisomerases are emerging in eukaryotes, such as activation of DNA damage response [128]; it would be thus of great relevance to determine the exact role of each topoisomerase in cellular processes of hyperthermophilic archaea, their association with transcription/replication machinery and their role in maintenance of genome stability.
